# H_2_S protects hippocampal neurons against hypoxia-reoxygenation injury by promoting RhoA phosphorylation at Ser188

**DOI:** 10.1038/s41420-021-00514-z

**Published:** 2021-06-04

**Authors:** Ye Chen, Jiyue Wen, Zhiwu Chen

**Affiliations:** Department of Pharmacology, School of Basic Medical Sciences, Anhui Medical University, Hefei, 230032 China

**Keywords:** Neuroscience, Neurological disorders

## Abstract

Inhibition of RhoA-ROCK pathway is involved in the H_2_S-induced cerebral vasodilatation and H_2_S-mediated protection on endothelial cells against oxygen-glucose deprivation/reoxygenation injury. However, the inhibitory mechanism of H_2_S on RhoA-ROCK pathway is still unclear. The aim of this study was to investigate the target and mechanism of H_2_S in inhibition of RhoA/ROCK. GST-RhoA^wild^ and GST-RhoA^S188A^ proteins were constructed and expressed, and were used for phosphorylation assay in vitro. Recombinant RhoA^wild^-pEGFP-N1 and RhoA^S188A^-pEGFP-N1 plasmids were constructed and transfected into primary hippocampal nerve cells (HNCs) to evaluate the neuroprotective mechanism of endothelial H_2_S by using transwell co-culture system with endothelial cells from cystathionine-γ-lyase knockout (CSE^−/−^) mice and 3-mercaptopyruvate sulfurtransferase knockout (3-MST^−/−^) rats, respectively. We found that NaHS, exogenous H_2_S donor, promoted RhoA phosphorylation at Ser188 in the presence of cGMP-dependent protein kinase 1 (PKG1) in vitro. Besides, both exogenous and endothelial H_2_S facilitated the RhoA phosphorylation at Ser188 in HNCs, which induced the reduction of RhoA activity and membrane transposition, as well as ROCK_2_ activity and expression. To further investigate the role of endothelial H_2_S on RhoA phosphorylation, we detected H_2_S release from ECs of CSE^+/+^ and CSE^−/−^ mice, and 3-MST^+/+^ and 3-MST^−/−^ rats, respectively, and found that H_2_S produced by ECs in the culture medium is mainly catalyzed by CSE synthase. Moreover, we revealed that both endothelial H_2_S, mainly catalyzed by CSE, and exogenous H_2_S protected the HNCs against hypoxia-reoxygenation injury via phosphorylating RhoA at Ser188.

## Introduction

Ischemic stroke is one of the main factors responsible for morbidity and death^1^. The restoration of blood flow is a very important way for the treatment of ischemic injury. However, the restoration of blood flow will cause reperfusion injury. Cerebral ischemia-reperfusion (I/R) can cause neuronal damage in varying degrees, including inflammation, necrosis, and apoptosis^2^. Cerebral I/R injury is particularly prone to induce apoptosis of vertebral neurons in the hippocampus area^3,4^. Therefore, the protection of hippocampal nerve cells (HNCs) is extremely important in the treatment of I/R injury. Previous researchers reported that the brain neurovascular unit (NVU) containing endothelial cells (ECs), HNCs, pericytes, astrocytes, and other cells regulates the metabolic homeostasis of the central nervous system and plays an important role in the neuroprotection and repair of the damaged tissue induced by ischemic stroke^5–8^. It must be pointed out that stabilization of EC function has a potential neuroprotective effect on the cerebral ischemic jury^9^.

Hydrogen sulfide (H_2_S) is a ubiquitous second messenger molecule. Endogenous H_2_S is mainly produced from l-cysteine by cystathionine-γ-lyase (CSE) and cystathionine-β-synthase, and from β-mercaptopyruvic acid catalyzed by 3-mercaptopyruvate sulfurtransferase (3-MST) in the mitochondria. In vasculature, endogenous H_2_S is mainly produced by CSE and 3-MST^10–13^. H_2_S has many important functions, including modifying neuronal conduction, regulating vascular tone, protecting tissues from oxidative stress, anti-oxidation, anti-apoptosis, and anti-inflammatory^14^. NaHS, an exogenous H_2_S donor, can also alleviate the neurotoxin acrylonitrile-induced neuronal damage in rats^15,16^ and inhibit rat retinal ganglion cell apoptosis^17^. Our previous studies have demonstrated that CSE-produced H_2_S has a protective effect on cerebral I/R injury in mice via inhibition of the RhoA/Rho-kinase (ROCK) pathway^18^.

RhoA, belonging to the small GTPases family, is widely distributed in the cytoplasm and is activated when it combines with GTP, and translates from the cytoplasm to the cell membrane. RhoA controls the activity of copious downstream effectors, such as ROCK^19^. ROCK contains two isoforms ROCK_1_ and ROCK_2_. ROCK_2_ is regarded as being responsible for the neuronal death and axon degeneration and regeneration after ischemic stroke^20^. Thus, the RhoA/ROCK pathway is closely related to the growth of neurons and axons^21,22^. Besides, emerging evidences indicated that the RhoA-ROCK pathway is involved in the pathological process of the central nervous system diseases. For instance, ischemic brain injury induces RhoA activation and upregulation of ROCK_2_ expression^23^, which leads to the neuronal cell death^24^, and inhibition of ROCK protects rats against hippocampal damage induced by cerebral I/R^25^. Although it has been confirmed that increase of H_2_S release has a protective effect on nerve cells against cerebral I/R injury via inhibition of the RhoA/ROCK pathway^18^, the target and mechanism of H_2_S in inhibition of RhoA/ROCK pathway is unknown and there is no research report on the relationship between H_2_S in the cerebral vascular ECs and RhoA-ROCK pathway in nerve cells.

The phosphorylation of RhoA at the Ser188 site can inhibit its activity and mediate its translocation from the cytoplasm to the cell membrane^26^. On the contrary, decreased phosphorylation of RhoA Ser188 in the hippocampal neuronal cells induces RhoA activation^27^. It has been reported that the phosphorylation of RhoA Ser188 mediated by cAMP or cGMP kinase can be regulated by many factors such as ste20-related kinase (SLK), angiotensin II type 2 receptor, and nitric oxide (NO)^28,29^. In view of the fact that H_2_S inhibits the activity of RhoA, we speculate that H_2_S may regulate the phosphorylation of RhoA at Ser188 such as NO, thereby protecting the brain against hypoxia-reoxygenation (H/R) injury.

In the present study, we first constructed and expressed wild-type GST-RhoA^wild^ and mutant GST-RhoA^S188A^ proteins in vitro, and carried out in vitro phosphorylation and autoradiography tests to explore the effect of exogenous H_2_S on RhoA phosphorylation. Next, we constructed and transfected the recombinant eukaryotic plasmids (RhoA^wild^-pEGFP-N1 and RhoA^S188A^-pEGFP-N1) into HNCs, to further analyse the effect of exogenous and endogenous H_2_S on the phosphorylation of RhoA at the Ser188, and the associated neuroprotective effects.

## Results

### Effect of H_2_S on RhoA Ser188 phosphorylation in vitro

To determine the effect of H_2_S on Ser188 phosphorylation of RhoA, GST-RhoA^wild^ and GST-RhoA^S188A^ proteins expressed in *Escherichia coli* were extracted and purified (Supplementary Fig. S1A–D, I–K), and were used for in vitro phosphorylation assay.

**Fig. 1:**
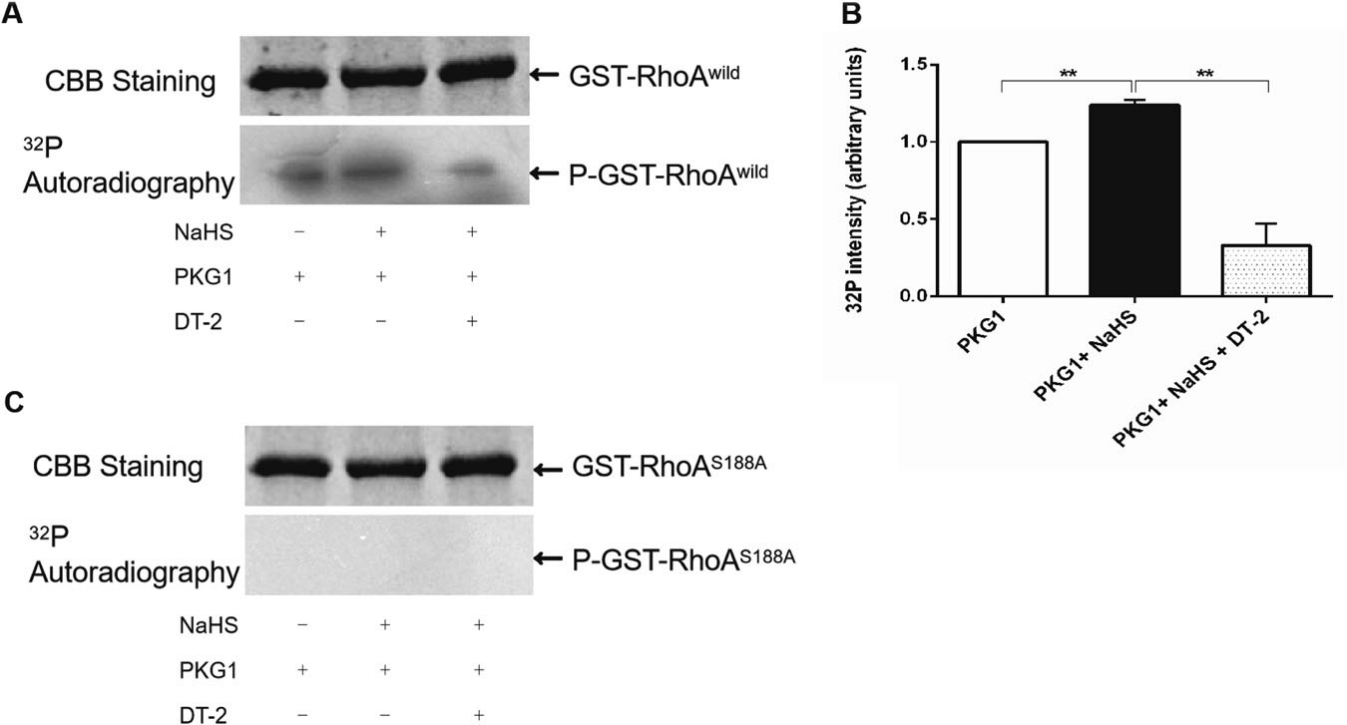
H_2_S phosphorylates RhoA on Ser188 in vitro. Representative image (**A**) and summary data (**B**) of NaHS phosphorylates GST-RhoA^wild^ tested by CBB staining and autoradiography. (**C**) Representative image of NaHS phosphorylates GST-RhoA^S188A^. Data are shown as the mean ± SEM, *n* = 3, **P < 0.01.

As shown in Fig. 1A, B, results of autoradiography showed that H_2_S donor NaHS (100 μmol/L) significantly promoted the phosphorylation of GST-RhoA^wild^ protein in the presence of protein kinase 1 (PKG1), but the promoted phosphorylation was significantly attenuated by PKG1 inhibitor DT-2 (2 μmol/L). However, Fig. 1C showed that there was no phosphorylation of GST-RhoA^S188A^ protein. These results indicated that H_2_S could promote phosphorylation of RhoA and Ser188 is required for the H_2_S-mediated RhoA phosphorylation.

### Ser188 mediated H_2_S-induced RhoA phosphorylation in rat hippocampal neuron

Effect of H_2_S on the RhoA phosphorylation and role of Ser188 in the RhoA phosphorylation were confirmed in cultured primary rat hippocampal neurons. As shown in Supplementary Fig. S2A–F, the green fluorescence caused by microtubule-associated protein 2 (MAP-2, a specific marker of neuron) antibody was presented in the cytoplasm of the cultured cells, suggesting that the cultured rat hippocampal cells were neurons.

**Fig. 2:**
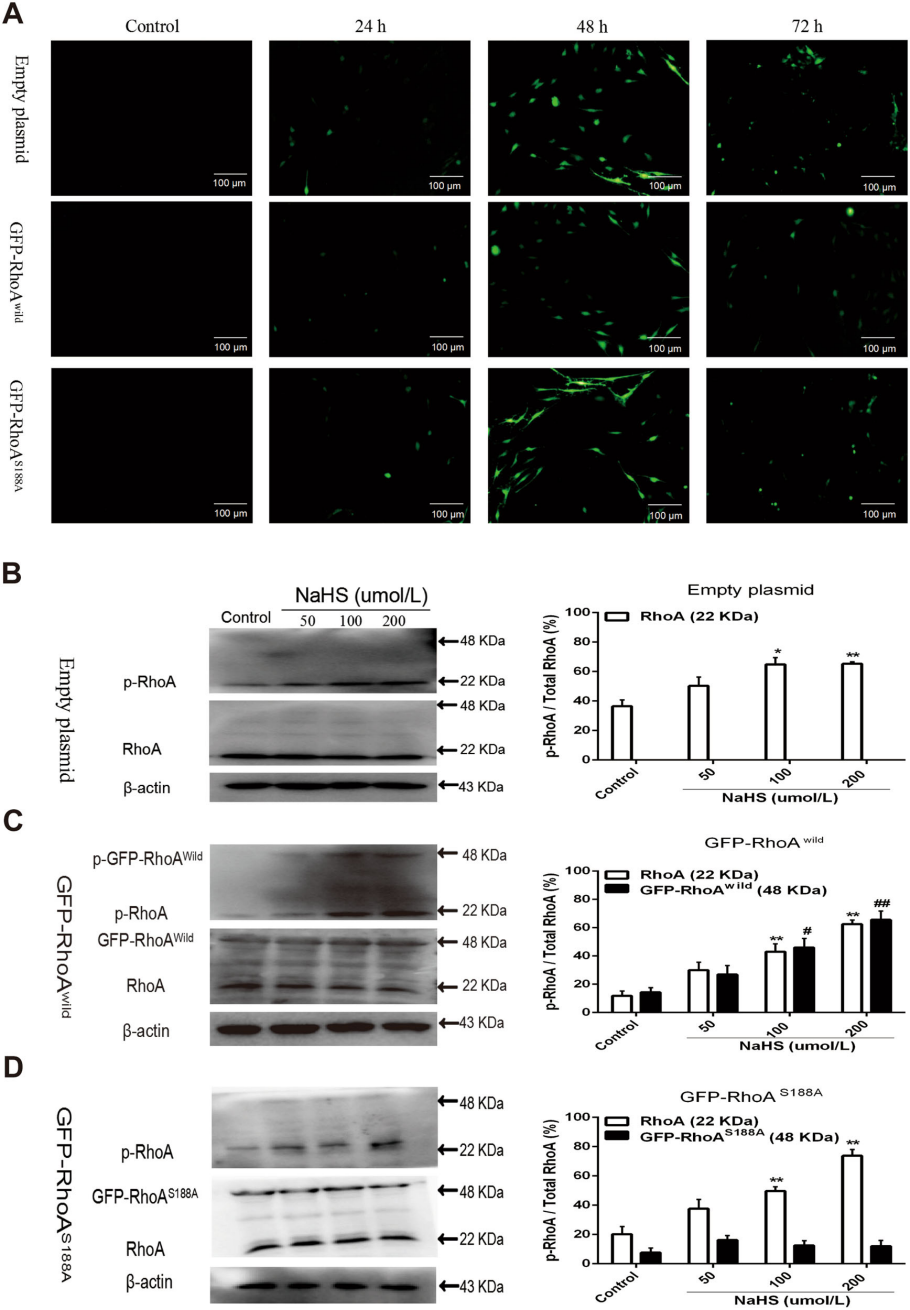
Ser188 mediated H_2_S-induced RhoA phosphorylation in HNCs. (**A**) Representative images of HNCs transfected with empty, GFP-RhoA^wild^, and GFP-RhoA^S188A^ plasmids, respectively (GFP-tagged, 100 μm). (**B**–**D**) Representative immunoblot images (left) and summary data (right) showing the effect of NaHS on p-RhoA expressions in HNCs. Here, 48 kDa refers to GFP-RhoA and p-GFP-RhoA; 22 kDa refers to RhoA and p-RhoA. Data are shown as the mean ± SEM; *n* = 3. *P < 0.05, **P < 0.01 or ^#^*P* < 0.05, ^##^*P* < 0.01 vs. the control.

The green fluorescent protein (GFP)-labeled wild-type RhoA (GFP-RhoA^wild^) and its mutant (GFP-RhoA^S188A^) eukaryotic expression plasmids pEGFP-N1 (Supplementry Fig. S1E–H) were respectively transfected into rat hippocampal neurons by using transient electrotransfection. Results showed that the transfection efficiency was significantly higher at 48 h after transfection than that at 24 h or at 72 h (Fig. 2A). Therefore, neurons transfected with either GFP-RhoA^wild^ or GFP-RhoA^S188A^ plasmids for 48 h were used for further experiment.

By using western blotting assay, untagged (RhoA), phosphorylated untagged RhoA (p-RhoA), GFP-RhoA^wild^, phosphorylated GFP-RhoA^wild^ (p-GFP-RhoA^wild^), GFP-RhoA^S188A^, and phosphorylated GFP-RhoA^S188A^ (p-GFP-RhoA^S188A^) were detected. As shown in Fig. 2B–D, treatment of NaHS at 100 and 200 µmol/L for 1 h promoted phosphorylation of RhoA in the neurons transfected with empty or GFP-RhoA^wild^, or GFP-RhoA^S188A^ plasmids, as well as phosphorylation of GFP-RhoA^wild^ in the GFP-RhoA^wild^ plasmid-transfected neurons, p-RhoA/RhoA ratio, or p-GFP-RhoA^wild^/GFP-RhoA^wild^ ratio increased markedly compared with those in the control group. However, NaHS had no effect on phosphorylation of GFP-RhoA^S188A^; p-GFP-RhoA^S188A^/GFP-RhoA^S188A^ ratio in the GFP-RhoA^S188A^ plasmid-transfected neuron did not significantly change in comparison with that in the control group. These results suggested that H_2_S could induce RhoA phosphorylation at Ser188 residue in rat hippocampal neurons.

### Role of Ser188 in H_2_S-induced RhoA translocation and inactivation in the neurons

**Fig. 3:**
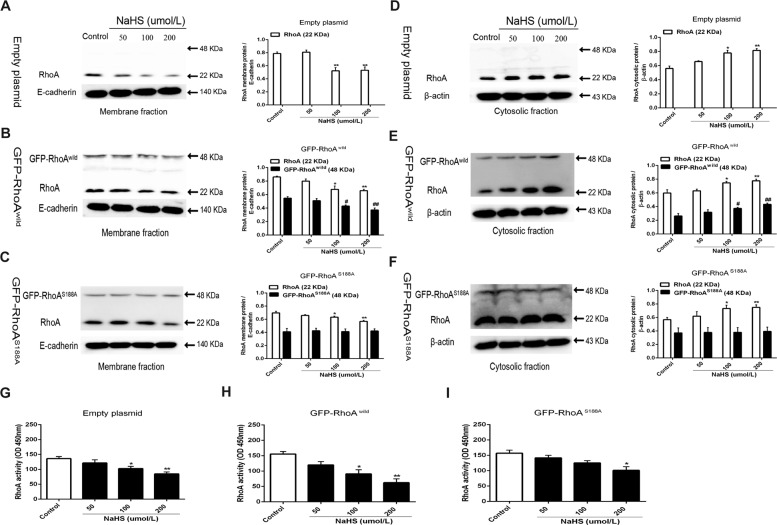
Role of Ser188 in H_2_S-induced RhoA translocation and inactivation in the neurons. (**A**–**C**) Representative immunoblot images (left) and summary data (right) showing the effect of NaHS on membrane fraction of RhoA in HNCs transfected with empty, GFP-RhoA^wild^, and GFP-RhoA^S188A^ plasmids, respectively. E-cadherin was used as a loading control. (**D**–**F**) Representative immunoblot images (left) and summary data (right) showing the effect of NaHS on cytosolic fraction of RhoA in HNCs transfected with empty, GFP-RhoA^wild^, and GFP-RhoA^S188A^ plasmids, respectively. β-Actin was used as a loading control. Here, 48 kDa refers to GFP-RhoA; 22 kDa refers to RhoA. Data are shown as the mean ± SEM; *n* = 3. *P < 0.05, **P < 0.01 or ^#^*P* < 0.05, ^##^*P* < 0.01 vs. the control. (**G**–**I**) NaHS inhibits RhoA activity in HNCs transfected with empty, GFP-RhoA^wild^, and GFP-RhoA^S188A^ plasmids, respectively. Data are shown as the mean ± SEM; *n* = 3. *P < 0.05 vs. the control.

Accumulating evidences have shown that when RhoA changes from an active state to an inactive state, it will translocate from the plasma membrane to the cytosol^27^. As shown in Fig. 3A–F, the results of western blotting showed that in primary rat hippocampal neurons transfected with either empty or GFP-RhoA^wild^, or GFP-RhoA^S188A^ plasmids, treatment of 100 and 200 µmol/L NaHS for 1 h caused significant decreases of the locations of RhoA and GFP-RhoA^wild^ in the plasma membrane, but promoted their location in the cytosol. However, 100 and 200 µmol/L NaHS had no obvious effect on location of GFP-RhoA^S188A^ in the plasma membrane and the cytosol. These results indicated that the Ser188 of RhoA is required for the H_2_S-caused translocation of RhoA from the plasma membrane to cytosol in the neuron.

Figure 3G–I showed that 100 and 200 µmol/L NaHS could significantly inhibit the RhoA activity in the neuron transfected with either empty plasmids or GFP-RhoA^wild^ plasmids, but this inhibitory effect was significantly decreased in the GFP-RhoA^S188A^ plasmid-transfected neurons, which was indicated by no inhibition of 100 µmol/L NaHS on RhoA activity. These results suggested that H_2_S could inhibit RhoA activity through Ser188.

### Role of RhoA Ser188 in H_2_S-induced inhibition of ROCK_2_ protein expression and its activity

**Fig. 4:**
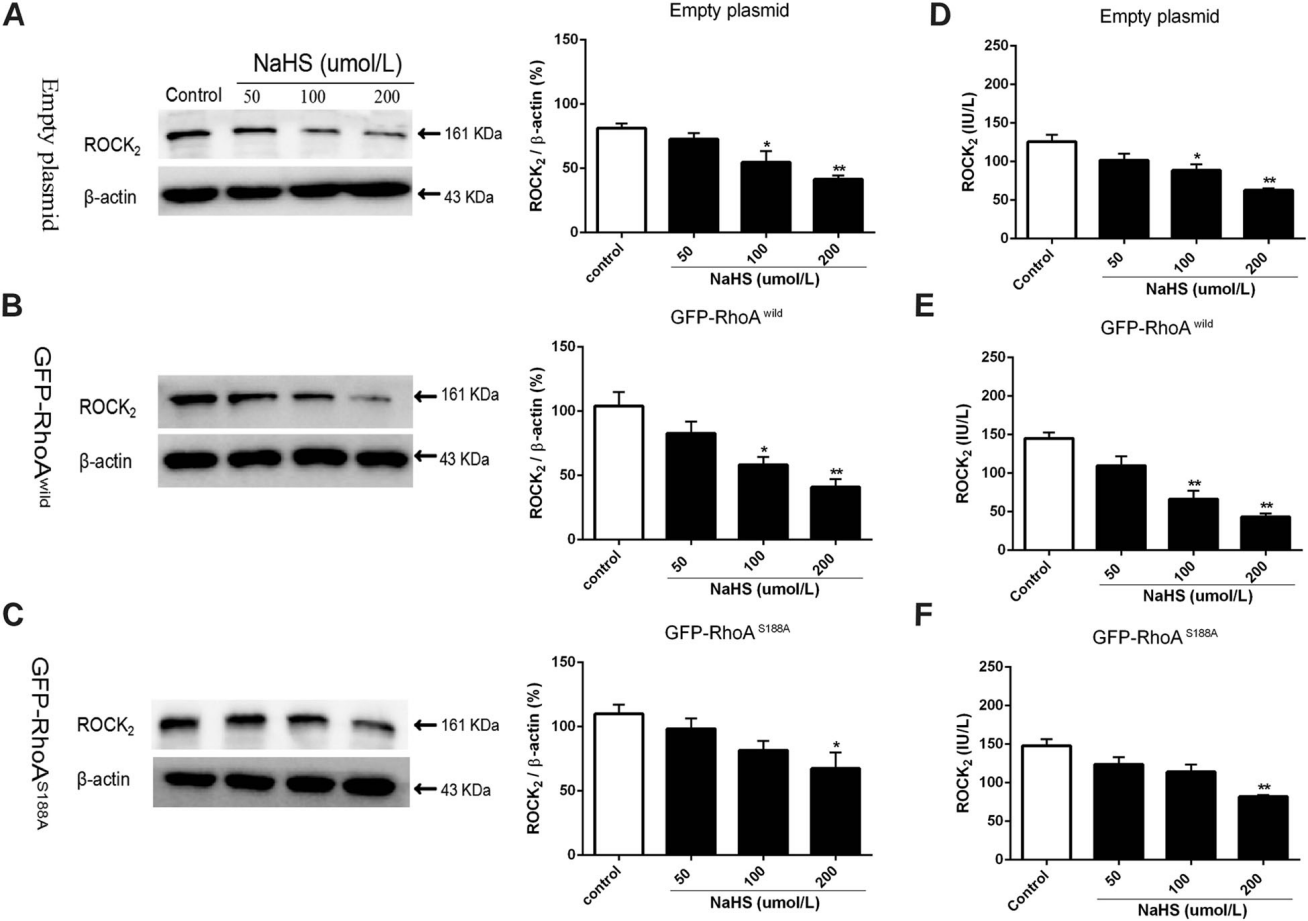
H_2_S-induced inhibition of ROCK_2_ protein expression and its activity. (**A**–**C**) Representative immunoblot images (in the left) and summary data (in the right) showing the effect of NaHS on ROCK_2_ expression in HNCs transfected with empty, GFP-RhoA^wild^, and GFP-RhoA^S188A^ plasmids, respectively. β-Actin was used as a loading control. (**D**–**F**) NaHS inhibits ROCK_2_ activity in HNCs transfected with empty, GFP-RhoA^wild^, and GFP-RhoA^S188A^ plasmids, respectively. Data are shown as the mean ± SEM; *n* = 3. *P < 0.05, **P < 0.01 vs. the control group.

As shown in Fig. 4, NaHS 100 and 200 µmol/L significantly inhibited the ROCK_2_ protein expression and its activity in rat hippocampal neurons transfected with either empty plasmids or GFP-RhoA^wild^ plasmids. However, in GFP-RhoA^S188A^ plasmid-transfected neurons, 100 µmol/L NaHS did not have this inhibitory effect and only 200 µmol/L NaHS had a significant inhibition. These results indicated that Ser188 of RhoA is associated with the H_2_S-induced inhibition of ROCK_2_ protein expression and its activity in the neurons.

### RhoA Ser188 mediated in protection of H_2_S on H/R injury in rat hippocampal neurons

**Fig. 5:**
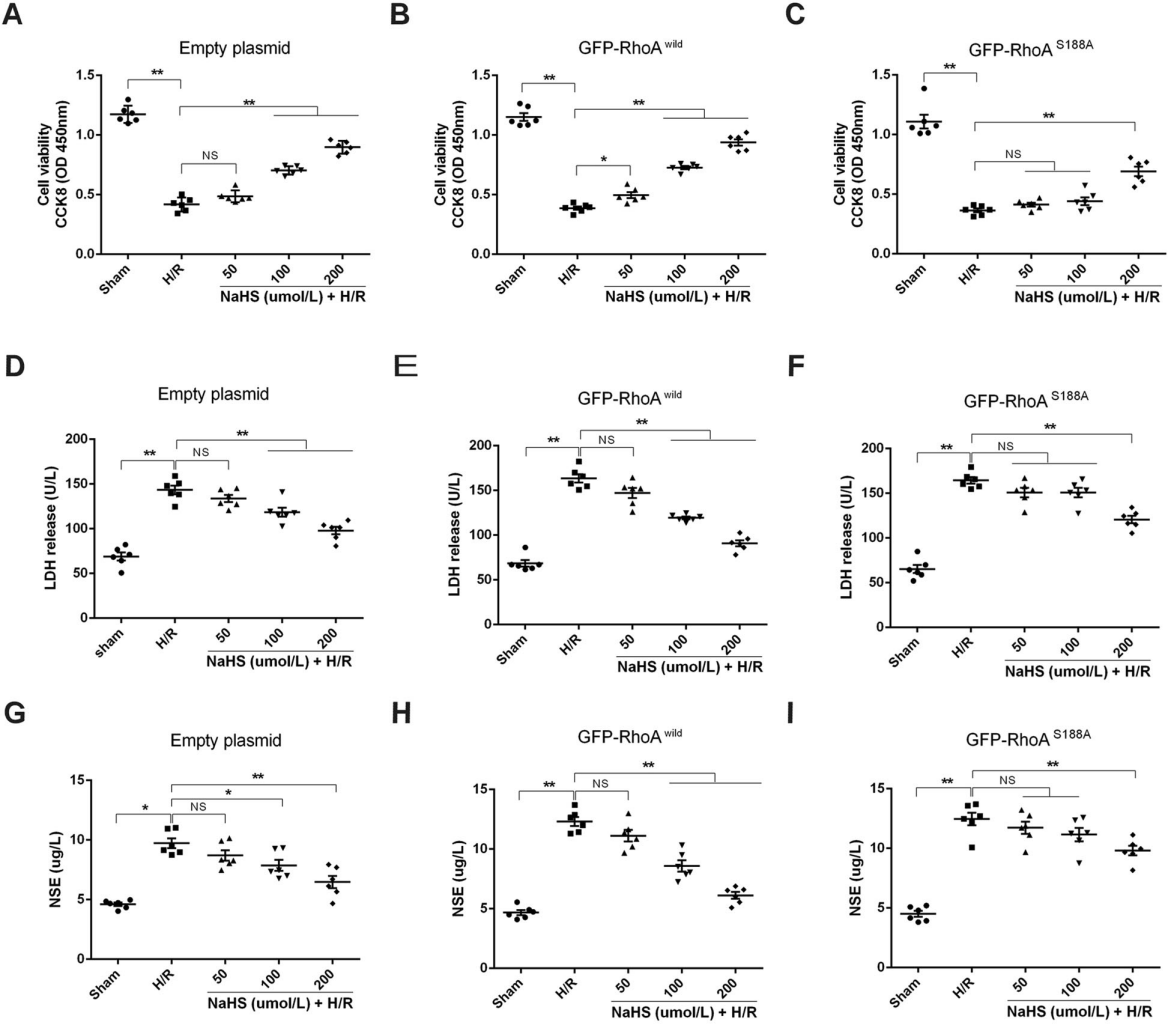
RhoA Ser188 mediated the protection of H_2_S on H/R injury in HNCs. (**A**–**C**) Effects of NaHS on cell viability of HNCs transfected respectively with empty, GFP-RhoA^wild^, and GFP-RhoA^S188A^ plasmids following H/R. (**D**–**F**) Effects of NaHS on lactate dehydrogenase (LDH) release from H/R injury HNCs transfected with empty, GFP-RhoA^wild^, and GFP-RhoA^S188A^ plasmids, respectively. (**G**–**I**) Changes of release of nerve-specific enolase (NSE) from H/R injury HNCs transfected with empty, GFP-RhoA^wild^, and GFP-RhoA^S188A^ plasmids, respectively. Data are shown as the mean ± SEM; *n* = 6. *P < 0.05, **P < 0.01.

As shown in Fig. 5, H/R injury in rat hippocampal neurons transfected with either empty plasmids or GFP-RhoA^wild^ plasmids, or GFP-RhoA^S188A^ plasmids, was indicated by the decreased cell viability and the increased activities of lactate dehydrogenase (LDH) and nerve-specific enolase (NSE) in the cultured supernatant. In the empty plasmid- or GFP-RhoA^wild^ plasmid-transfected neurons, both 100 and 200 μmol/L NaHS significantly inhibited the decreased cell viability and the increased activities of LDH and NSE. In addition, 50 µmol/L NaHS also inhibited the decreased cell viability in the GFP-RhoA^wild^ plasmid-transfected neurons. However, inhibitory effects of NaHS were markedly reduced in GFP-RhoA^S188A^ plasmid-transfected neurons, only up to 200 µmol/L; NaHS significantly inhibited the decreased cell viability and the increased activities of LDH and NSE. These results indicated that RhoA Ser188 mediated the protective effect of H_2_S on H/R injury.

### Effect of endothelial-derived H_2_S on RhoA phosphorylation, activity, and translocation in the neurons

By using co-culture of primary cerebrovascular ECs with rat hippocampal neurons, effects of endothelial-derived H_2_S on phosphorylation, activity, and translocation of RhoA in the neurons were studied. Due to the fact that CSE and 3-MST are the main enzymes responsible for the synthesis of H_2_S in vascular ECs^10–13^, genetic identification of CSE^−/−^ and CSE^+/+^ mice, as well as 3-MST^+/+^ and 3-MST^−/−^ rats was undertaken (Supplementary Fig. S3A, B). Cerebrovascular ECs isolated from CSE wild-type and knockout mice or 3-MST wild-type and knockout rats were identified by factor VIII (Supplementary Fig. S2G–L) and were used in co-culture, and 1 × 10^−6^ mol/L acetylcholine (ACh) was used to stimulate H_2_S release from ECs.

As shown in Supplementary Fig. S4A–D, western blotting results showed that p-RhoA/total RhoA ratio and RhoA in the cytosol were significantly reduced, whereas RhoA activity and RhoA location in the plasma membrane were markedly increased in the neurons co-cultured with CSE^−/−^ ECs compared with those in the neurons co-cultured with CSE^+/+^ ECs. However, endothelial 3-MST knockout did not affect p-RhoA/total RhoA ratio, RhoA activity, and RhoA distribution in the neurons co-cultured with ECs. These results suggested that endothelial-derived H_2_S could induce phosphorylation of RhoA, inhibited its activity, and caused translocation of RhoA from the plasma membrane to the cytosol in rat hippocampal neurons, and perhaps CSE-produced H_2_S exerted a predominant effect.

### Effect of endothelial-derived H_2_S on ROCK_2_ protein expression and activity in the neurons

As shown in Supplementary Fig. S5A, B, both ROCK_2_ protein expression and activity significantly increased in neurons co-cultured with CSE^−/−^ ECs compared with those in the neurons co-cultured with CSE^+/+^ ECs. However, endothelial 3-MST knockout in ECs (3-MST^−/−^ ECs) had no effect on ROCK_2_ protein expression and activity in the co-cultured neurons. The results indicated that endothelial CSE-produced H_2_S could significantly inhibit ROCK_2_ expression and activity in the neurons_._

### Endothelial CSE-produced H_2_S in co-culture model of ECs and the neuron

Supplementary Fig. S5C showed that H_2_S content in the culture supernatant significant lowered in co-culture model of CSE^−/−^ ECs and neurons than that in co-culture model of CSE^+/+^ ECs and neurons. However, 3-MST knockout in ECs had no effect on H_2_S content in the culture supernatant of co-culture model. The results suggested that endothelial-derived H_2_S in the culture supernatant was mainly produced by CSE rather than 3-MST.

### Role of Ser188 in CSE-produced H_2_S-induced RhoA phosphorylation, translocation, and ROCK_2_ protein expression in rat neurons

In the co-culture of rat hippocampal neurons and CSE^+/+^ ECs or CSE^−/−^ ECs, the neurons were transfected with empty plasmids or GFP-RhoA^wild^ plasmids, or GFP-RhoA^S188A^ plasmids.

**Fig. 6:**
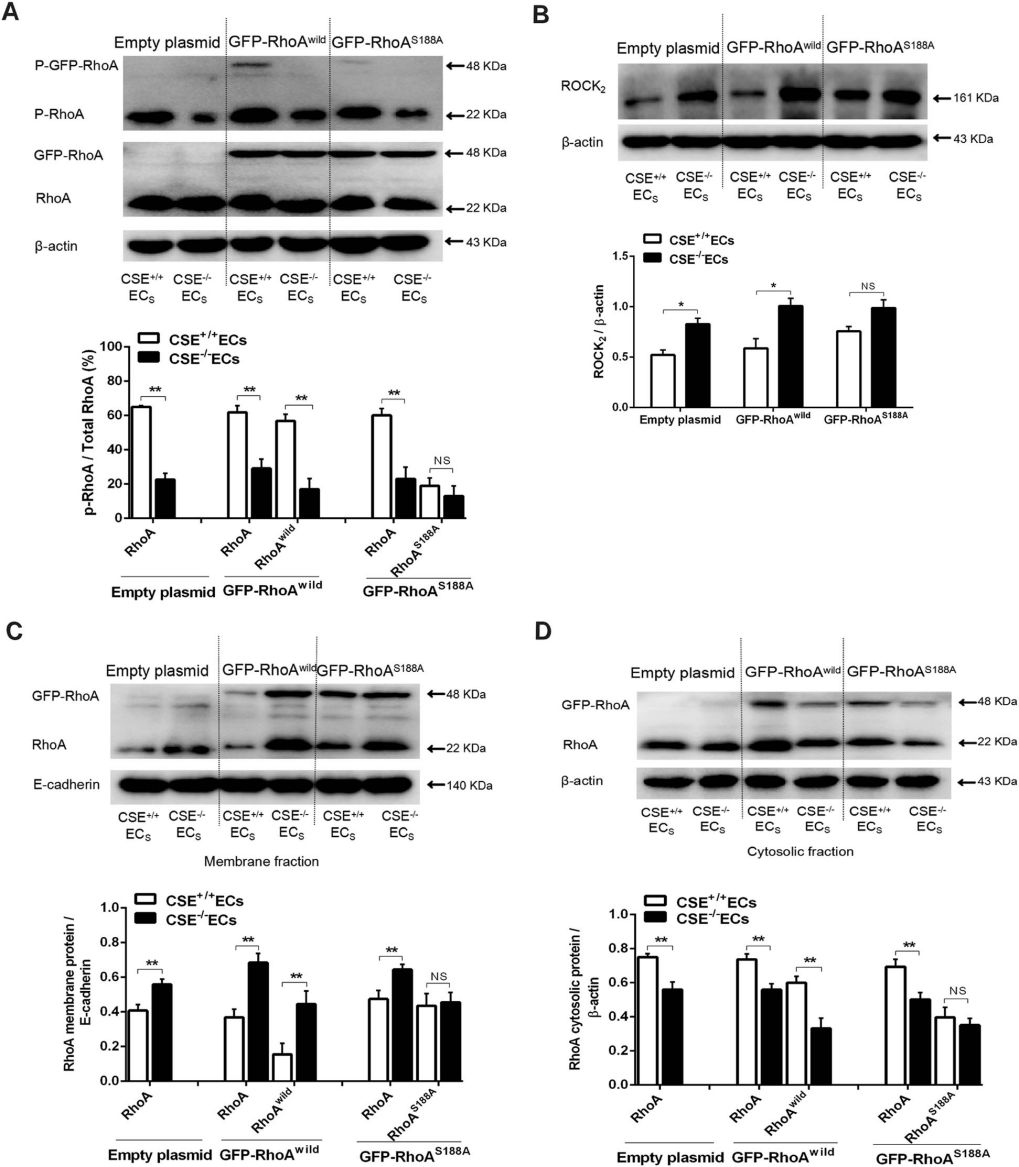
CSE-produced H_2_S-induced changes of RhoA phosphorylation, transloction, and ROCK_2_ protein expression in HNCs. Expressions of p-RhoA (**A**) and ROCK_2_ (**B**) in HNCs. β-Actin was used as a loading control. RhoA expression in membrane fraction (**C**) and cytosolic fraction (**D**) of HNCs, which were transfected with empty, GFP-RhoA^wild^, or GFP-RhoA^S188A^ plasmids, and co-cultured with endothelial cells derived from CSE^−/−^ or CSE^+/+^ mice. Here, 48 kDa and 22 kDa refer to GFP-RhoA and RhoA, respectively. Data are shown as the mean ± SEM; *n* = 3. *P < 0.05, **P < 0.01.

As shown in Fig. 6A, p-RhoA/RhoA ratio in empty or GFP-RhoA^wild^, or GFP-RhoA^S188A^ plasmid-transfected neurons co-cultured with CSE^−/−^ ECs was markedly lowered than that in neurons co-cultured with CSE^+/+^ ECs. Similarly, co-culture with CSE^+/+^ ECs has enhanced effect on the p-GFP-RhoA^wild^/GFP-RhoA^wild^ ratio in the neurons transfected with GFP-RhoA^wild^ plasmids, but had no effect on the ratio of p-GFP-RhoA^S188A^/GFP-RhoA^S188A^ in the neurons transfected with GFP-RhoA^S188A^ plasmids. The results suggested that Ser188 mediated RhoA phosphorylation in rat hippocampal neurons induced by endothelial CSE-produced H_2_S. Besides, Fig. 6A also showed the weak band of p-GFP-RhoA (48 kDa) from neurons transfected with GFP-RhoA^S188A^ and co-cultured with CSE^+/+^ ECs, suggesting the H_2_S-induced RhoA phosphorylation in different residue at a low level.

Figure 6B–D showed that knockout of CSE in ECs significantly decreased RhoA location in the cytosol, increased RhoA distribution in the membrane, and ROCK_2_ protein expression in the co-cultured neurons transfected with empty or GFP-RhoA^wild^, or GFP-RhoA^S188A^ plasmids. In the co-cultured neurons transfected with GFP-RhoA^wild^ plasmids, the knockout of CSE induced a reduced GFP-RhoA^wild^ in the cytosol and increased GFP-RhoA^wild^ in membrane and ROCK_2_ protein expression. However, in the co-cultured neurons transfected with GFP-RhoA^S188A^ plasmids, the knockout of CSE did not affect the distribution of GFP-RhoA^S188A^ in the membrane and cytosol, as well as ROCK_2_ protein expression. The results suggested that endothelial CSE-produced H_2_S could induce translocation of RhoA from the membrane to cytosol and inhibition of ROCK_2_ protein expression in rat hippocampal neurons, and these effects are associated with Ser188 of RhoA.

### Role of Ser188 in protection of endothelial CSE-produced H_2_S against H/R injury in the neurons

**Fig. 7:**
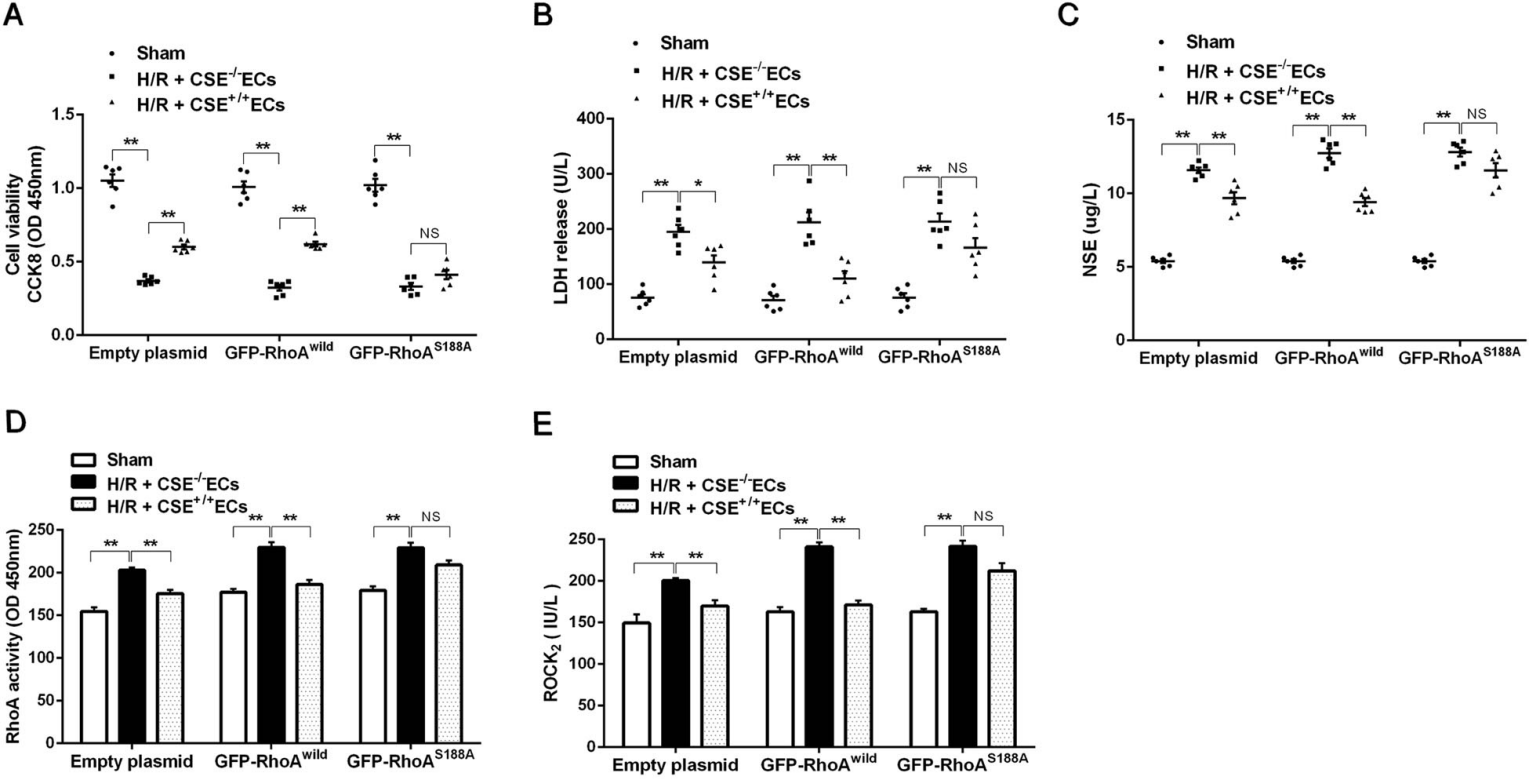
H_2_S produced by CSE in endothelial cells mitigates H/R injury and regulates the activity of RhoA and ROCK_2_ in HNCs. **(A**–**C)** Effects of CSE-produced H_2_S from endothelial cells on cell viability, release of LDH, and NSE from HNCs transfected with empty, GFP-RhoA^wild^, or GFP-RhoA^S188A^ plasmids under H/R conditions. Effects of endothelial CSE-produced H_2_S on RhoA activity (**D**) and ROCK_2_ activity (**E**) in HNCs transfected with empty, GFP-RhoA^wild^, or GFP-RhoA^S188A^ plasmids under H/R conditions. Data are shown as the mean ± SEM; *n* = 3. *P < 0.05, **P < 0.01.

As shown in Fig. 7A–C, in CSE^−/−^ EC-co-cultured rat hippocampal neurons transfected with either empty plasmids or GFP-RhoA^wild^ plasmids, or GFP-RhoA^S188A^ plasmids, 8 h of hypoxia followed by 6 h of reoxygenation induced a significant H/R injury indicated by decreased cell viability and increased activities of LDH and NSE in cultured supernatant compared with those in the sham group. H/R-induced cell injury was markedly decreased in CSE^+/+^ EC-co-cultured neurons transfected with either empty or GFP-RhoA^wild^ plasmids. However, co-cultured neurons transfected with GFP-RhoA^S188A^ plasmids had no effect on the H/R-induced reduction of cell viability and increases of LDH and NSE activities (Fig. 7D, E). The activity of RhoA and ROCK_2_ in the neurons were significantly increased following H/R injury. CSE^+/+^ ECs significantly decreased RhoA and ROCK_2_ activities in the co-cultured neurons transfected with empty or GFP-RhoA^wild^ plasmids, but had no effect in the co-cultured neurons transfected with GFP-RhoA^S188A^ plasmids. The results indicated that endothelial CSE-produced H_2_S could inhibit the activity of RhoA and ROCK_2_ in the neurons, and this effect is related to RhoA Ser188.

**Fig. 8:**
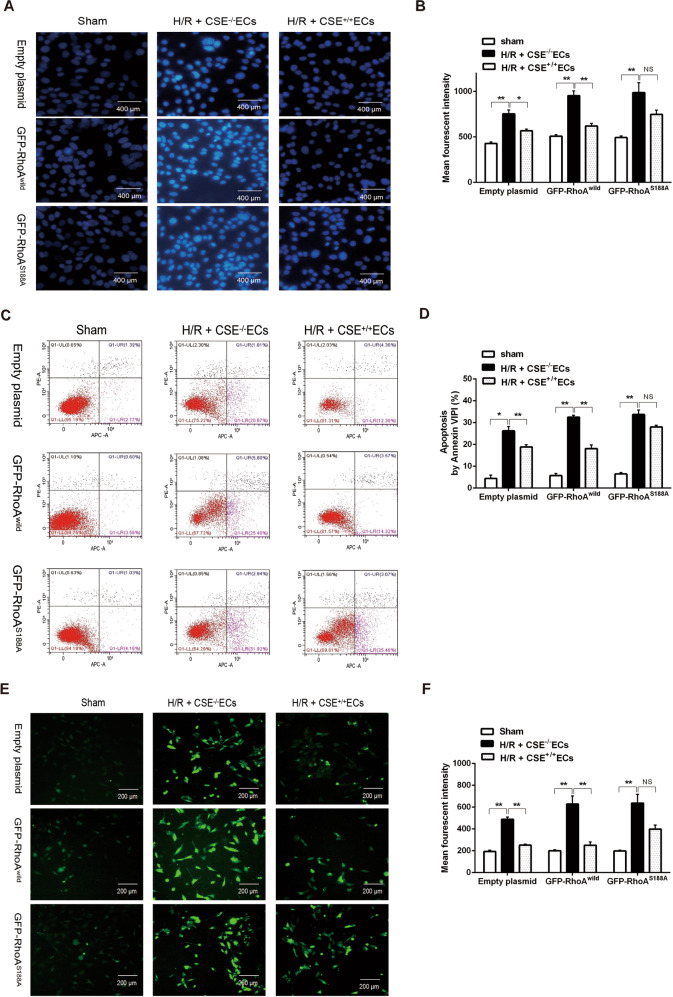
Role of Ser188 in the protection of endothelial CSE-produced H_2_S against H/R injury in the neurons. Hoechst 33258 staining, 400 μm; Fluo-8 staining, 200 μm. (**A**) Hoechst staining of apoptotic nucleus in HNCs transfected with empty, GFP-RhoA^wild^, or GFP-RhoA^S188A^ plasmids under hypoxia-reoxygenation (H/R) condition. Representative immunoblot images (**C**) and summary data (**B**, **D**) of cell apoptosis in HNCs transfected with empty, GFP-RhoA^wild^, or GFP-RhoA^S188A^ plasmids under H/R condition. Representative immunoblot images (**E**) and summary data (**F**) of calcium concentration in HNCs transfected with empty, GFP-RhoA^wild^, or GFP-RhoA^S188A^ plasmids under H/R condition. Data are shown as the mean ± SEM; *n* = 3. *P < 0.05, **P < 0.01.

Apoptosis is also a critical index for H/R injury. Hoechst staining and Annexin V/propidium iodide (PI) staining were respectively used for the detection of apoptotic cells in the present study. Figure 8A–F showed that H/R injury induced significant increases of apoptotic cells and intracellular free Ca^2+^ fluorescence intensity in CSE^−/−^ EC-co-cultured neurons transfected with either empty plasmids or GFP-RhoA^wild^ plasmids, or GFP-RhoA^S188A^ plasmids. CSE^+/+^ ECs could significantly decrease H/R injury-induced increase of apoptotic cells and intracellular free Ca^2+^ fluorescence intensity in co-cultured neurons transfected with empty or GFP-RhoA^wild^ plasmids, but had no effect in co-cultured neurons transfected with GFP-RhoA^S188A^ plasmids. These results suggested that endothelial CSE-produced H_2_S could protect the neuron from H/R injury via inhibiting the increase of intracellular free Ca^2+^ and the effects were mediated by RhoA Ser188.

## Discussion

In view of the condition that the phosphorylation of RhoA requires cAMP-dependent protein kinase A or cGMP-dependent PKG^30^, we used PKG1 as the kinase to catalyze the phosphorylation of RhoA at Ser188 in vitro. Our results showed that RhoA is phosphorylated in the presence of PKG1, which is consistent with the previous results that the gas signal molecule NO can phosphorylate RhoA at Ser188^31,32^. H_2_S is considered to be the third gas signal molecule and its physiological functions are receiving increasing attentions^33^. The pharmacological effects of H_2_S produced by NaHS are now considered reliable^34^. Our in vitro phosphorylation and autoradiography results showed that exogenous H_2_S could also promote the phosphorylation of RhoA at Ser188 mediated by PKG1, which was blocked by PKG1 inhibitors. In addition, the eukaryotic recombinant plasmids containing expressed genes of GFP-RhoA^wild^ and GFP-RhoA^S188A^ were respectively transferred into primary hippocampal neurons, to further confirm the effect of H_2_S. The results showed that NaHS increased the phosphorylated protein of RhoA^wild^, but not RhoA^S188A^ one in neurons by using western blotting. These findings revealed that H_2_S promoted phosphorylation of RhoA at Ser188; moreover, we also demonstrated that endothelial H_2_S, mainly produced by CSE, regulates RhoA phosphorylation at Ser188.

It is widely recognized that phosphorylation of RhoA at Ser188 directly inhibits RhoA activity^35^, but phosphorylation at Ser88 increases its activity^36^. In the present study, we demonstrated that RhoA activity is significantly inhibited in HNCs after phosphorylation at Ser188, which is consistent with previous results^37^. As we all know, the activated RhoA is mainly distributed in the cell membrane and non-activated RhoA is mainly distributed in the cytoplasm^38^, and NO increases the distribution of RhoA in the cytoplasm and reduces its location in the cell membrane, by promoting the phosphorylation of RhoA at Ser188. Besides, prostaglandin E1 may prevent RhoA translocation to the membrane by promoting the phosphorylation of RhoA at Ser188 on the platelet actin cytoskeleton^31,39^. Furthermore, in vascular smooth muscles, PKG-dependent inactive RhoA accumulates in the cytoplasm^40^. Our results also showed that the distribution of RhoA in neuronal membranes is significantly reduced and its distribution in the cytoplasm is significantly increased after RhoA phosphorylation induced by H_2_S. Previous researches have reported that RhoA activity can not only affect its own translocation but also regulate the activity and expression of its downstream signaling molecule ROCK_2_^41–43^. Phosphorylation of RhoA at Ser188 prevents the activation of RhoA and reduces the affinity between RhoA with ROCK^44,45^. In this study, we also found that both ROCK_2_ activity and expression were significantly inhibited in primary hippocampal neurons, when RhoA was phosphorylated in Ser188.

The phosphorylation and dephosphorylation of proteins play crucial roles in the regulation of synaptic function under physiological and pathophysiological conditions^46^. For example, phosphorylation of RhoA at Ser188 mediated by protein kinase A prevents it from binding to ROCK and then promotes neuronal differentiation and synaptic proliferation^47^. Furthermore, phosphorylation of RhoA is involved in the protection of ischemic damage of the heart, brain, etc.^48^. Accumulating studies have shown that ischemic brain injury induces a significant decrease of nerve cell viability and increases of LDH and NSE leakages. Hence, similar to cell viability, LDH and NSE leakages have also been applied to assess neuron injury^49,50^. Other researchers reported that H_2_S is predicted to have physiological functions in the brain^51^. We found that H/R induces significant increases of LDH and NSE leakages, and reduction of neuronal viability, which could be inhibited by NaHS. However, the inhibitory effects of NaHS were markedly reduced in GFP-RhoA^S188A^ plasmid-transfected neurons. These data indicated that exogenous H_2_S reduces hippocampal neuronal damage induced by H/R, by promoting RhoA phosphorylation at Ser188.

As aforementioned, the homeostasis of the central nervous system is maintained by the brain NVU^52^. Therefore, the co-culture model of ECs and nerve cells was chosen to further demonstrate the role of endogenous H_2_S released from vascular ECs on the phosphorylation of RhoA at Ser188 in HNCs, membrane translocation of RhoA, ROCK_2_ expression, and activity. Moreover, the effect of endothelial H_2_S-induced RhoA phosphorylation on the damage of HNCs induced by hypoxia and reoxygenation was assessed. Our data revealed that H_2_S released from ECs could protect nerve cells against hypoxia and reoxygenation injury via promoting the RhoA phosphorylation at Ser188, which was supported by the results that CSE^+/+^ ECs could protect the neurons transfected with GFP-RhoA^wild^ but not GFP-RhoA^S188A^ against the hypoxia and reoxygenation injury, and inhibit the increase of intraneuronal Ca^2+^ in co-culture mode. These findings were consistent with previous research that H_2_S reduces the calcium ions in cells and protects myoblasts from apoptosis and oxidative stress induced by Golgi stress^53^. On the contrary, cerebral I/R-induced calcium overload in nerve cells is the promoter of nerve cell apoptosis^54^. Thus, our results indicated that the reduction of calcium ion concentration in cells may be involved in the RhoA phosphorylation at Ser188-induced neuroprotection of endothelial H_2_S. In a word, we demonstrated for the first time that both endothelial-derived H_2_S and exogenous H_2_S protect nerve cells from hypoxia and reoxygenation damage by promoting RhoA phosphorylation at Ser188.

Although endothelial H_2_S is mainly generated by CSE and 3-MST, our results showed that ACh-stimulated release of H_2_S from vascular ECs is mainly from CSE. The reason of CSE- and 3-MST-caused different amounts of H_2_S release is worthy of further exploration.

In summary, we demonstrated that exogenous and endothelial H_2_S promote RhoA phosphorylation at Ser188 for the first time. Our results also indicated that the RhoA phosphorylation at Ser188 contributes to inhibition of RhoA activity and membrane transposition, and reduction of ROCK_2_ activity and expression. Furthermore, we found that exogenous and endothelial H_2_S mitigates H/R injury of neurons via accelerating RhoA phosphorylation at Ser188. These findings provide potential targets for treating or preventing cerebral I/R injury.

## Material and methods

### Reagents

NaHS was obtained from Sigma Chemical (St. Louis, USA); [γ-^32^P] ATP was obtained from China Tongfu Co., Ltd (Beijing, China); PKG1, ATP, DT-2, and ACh were obtained from Sigma-Aldrich (St. Louis, MO, USA); LDH test kit was obtained from Nanjing Jiancheng Biotechnology (Nanjing, China); NSE Assay Kit was obtained from Jiangsu Meimian Industrial, Co., Ltd (Jiangsu); Fluo-8 AM, anti-ROCK_2_ (catalog number ab125025), anti-Phospho-RhoA Ser188 (catalog number ab41435), and anti-RhoA (catalog number 187027) were purchased from Abcam (San Francisco, California, USA); and anti-β-actin (catalog number AF7018) and goat anti-rabbit IgG secondary antibody (catalog number S0001) were purchased from Affinity Biosciences (Changzhou, China).

### Animal

Wild-type (CSE^+/+^) and CSE-knockout (CSE^−/−^) C57BL/6J mice, and wild-type (3-MST^+/+^) and 3-MST-knockout (3-MST^−/−^) Sprague Dawley (SD) rats were obtained from Shanghai Biological Model Biotechnology, Co., Ltd. The animals were housed in the Animal Center of Anhui Medical University, with free access to food and water, and controlled temperature at 22 ± 2 °C. All the efforts were undertaken to minimize the pain or discomfort of the animals; all experimental procedures were carried out in accordance with the operating procedures approved by the Ethical Review Committee of Anhui Medical University, which comply with the guidelines for the care and use of laboratory animals of the National Institutes of Health (NIH publication number 85-23, revised in 2011).

### Expression the recombinant RhoA and RhoA^S188A^

The glutathione-*S*-transferase (GST)-tag RhoA^wild^-pGEX-6p1 and RhoA^S188A^-pGEX-6p1 plasmids were constructed by Gene Create Biological Engineering, Co. (Wuhan, China) from rat genome. RhoA^S188A^ is that Ser188 of RhoA was mutated to an Ala at the mRNA level. The plasmids were expressed in *E. coli* and purified as previously described^26^. Two prokaryotic expression plasmids were transferred into BL-21 *E. coli*, respectively, and then the expressions of GST-RhoA^wild^ and GST- RhoA^S188A^ proteins were induced by isopropylthio-β-d-galactoside. The collected protein was sonicated and purified by GST-Sepharose 4B affinity chromatography (GST-Sepharose FF). We used the bicinchoninic acid method to quantitatively analyze the purified protein. The eluted GST-RhoA^wild^ and GST- RhoA^S188A^ proteins were used for in vitro phosphorylation assay.

### In vitro phosphorylation assay

In vitro phosphorylation assay was performed as previously described^26^. Briefly, the kinase buffer containing 50 mM Tris, 10 mM MgCl_2_, 1 mM dithiothreitol, and 100 μM ATP was prepared. Then, 20 μg of RhoA^wild^ or RhoA^S188A^, 10 μCiof [γ-^32^P]-ATP, 100 ng kinase and 100 μM NaHS were sequentially added to the kinase buffer and the mixture was then incubated in a shaker at 30 °C for 30 min. After that, the mixture was separated by SDS-polyacrylamide gel electrophoresis (SDS-PAGE) and stained by using Coomassie Brilliant Blue. The separated proteins were incubated with X-ray film for 72 h and displayed by autoradiography.

### EC culture

Primary rat brain vascular ECs were respectively isolated from 6 to 8 weeks old CSE^−/−^ and CSE^+/+^mice, and 3-MST^−/−^ and 3-MST^+/+^ rats, as previously described^55^. In short, rats were anesthetized by isoflurane. The intact brains were carefully isolated after cervical dislocation and placed in a culture dish with sterile phosphate buffered saline (PBS). The rat basilar artery was carefully separated and transferred to eppendorf tubes containing 0.1% type I collagenase and were quickly cut into pieces; vascular fragments were digested for 30 min at 37 °C. The mixture was centrifuged at 1000 r.p.m. for 10 min to obtain the cell pellet, which was resuspended in ECM medium (5% fetal bovine serum + 1% EC growth supplement + 1% penicillin–streptomycin). According to the principle of differential adhesion, the medium was changed after 1 h to obtain vascular ECs. The cells were identified by immunofluorescence staining for VIII.

### Neuronal culture

Primary rat hippocampal neurons were isolated from newborn SD rats. Briefly, newborn rats were soaked in 75% alcohol for surface disinfection under anesthesia, the brain was completely isolated after killing, and then the hippocampus tissue was separated and cut into pieces in 0.125% trypsin solution. After digestion for 20 min at 37 °C with being gently shaken every 5 min, the equal volume of complete medium was added to stop the digestion. Cells were collected after filtration and centrifugation, and Dulbecco’s modified Eagle complete medium was added to resuspend the culture cells in a six-well plate precoated with polylysine. The cells were incubated at 37 °C in 5% CO_2_ incubator. Twenty-four hours later, the medium was replaced with neurobasal serum-free medium (neurobasal + 2% B27 + 0.5 mmol/L l-glutamine + 1% penicillin–streptomycin); 48 h later, 20 μM cytarabine was used to inhibit the growth of glial cells. Immunofluorescence staining was performed to identify HNCs with neuronal MAP-2.

### Electroporation of eukaryotic expression plasmids of recombinant RhoA^wild^ and RhoA^S188A^ into cultured neurons

The GFP-tag eukaryotic expression plasmids containing RhoA^wild^- and RhoA^S188A^-expressed genes were constructed by Gene Create Biological Engineering, Co. (Wuhan, China) from rat genome. The two recombinant plasmids were designated as RhoA^wild^-pEGFP-N1 and RhoA^S188A^-pEGFP-N1, respectively. According to previous research^56^, ~5 × 10^6^ HNCs were mixed with 5 μg RhoA^wild^-pEGFP-N1 or RhoA^S188A^-pEGFP-N1 and 70~80 μL Opti-MEM. The mixed solution was transferred to the electroporation cuvette and electroporation was performed by using CUY21EDIT II super multi-pulse in vivo/cell electroporation instrument (BEX, Tokyo, Japan) with program of perforation pulse voltage 350 V, pulse drive voltage 20 V, pulse time 10 ms, and cycle number 20 times. Fluorescence microscope was used to detect transfection efficiency.

### H/R protocol

HNCs were cultured with sugar-free medium in a three-gas incubator (1% oxygen, 5% nitrogen, 94% carbon dioxide, 37 °C) for 8 h, then the cells were replaced with normal culture medium and transferred to the incubator under normal condition for 6 h. The control group cells are maintained under normal condition.

### Determination of RhoA and ROCK_2_ activities

Determination of RhoA and ROCK_2_ activities was performed according to the manufacturer’s instructions and our previous study^18^. Briefly, the lysate of HNCs was collected after cell sonication and centrifugation to measure RhoA and ROCK_2_ activities by G-LISA activation assay. Optical density (OD) value of the sample at 450 nm was recorded by using colorimetric assay.

### Intracellular calcium concentration ([Ca^2+^]_*i*_)

The cells were incubated with 10 μM Fluo-8 AM at 37 °C for 10 min. Then the cells were washed with NPSS (NaCl 140 mM, glucose 10 mM, KCl 5;mM, Hepes 5 mM, CaCl_2_ 1 mM, and MgCl_2_ 1 mM) solution. Then, 488 nm excitation was used. Intracellular Ca^2+^ signal was captured by using fluorescence microscope (Olympus IX73, Lambda DG-4 Sutter Instrument Company Novato America).

### Determination of cell viability and LDH

Cell viability and LDH activity in cell culture supernatant were determined according to the manufacturer’s instructions. Briefly, cultured cells were incubated with 10 μl cell counting kit-8 solution for 1 h. Cell activity was detected at 450 nm by a microplate reader. The cell culture medium was collected to detect the LDH activity by using spectrophotometry at 450 nm and the result was expressed as U/L.

### Determination of NSE activity

NSE activity in cell-cultured supernatant was assessed according to the manufacturer’s instructions. Supernatant of cell culture was collected and transferred to 96-well plates to detect the NSE activity by using rat NSE enzyme-linked immunoassay kit. The OD value at 450 nm was recorded by a microplate reader and the results were expressed as μg/L.

### Flow cytometry assay for the cell apoptosis

Annexin V-Alexa Fluor 647/PI cell apoptosis detection kit was used to detect cell apoptosis in compliance with the manufacturer’s instructions. The cells were collected and washed with PBS, and then resuspended in binding buffer. After that, cells were incubated in PI and fluorescein isothiocyanate-Annexin V for 30 min. The percentage of apoptotic cells was analyzed on flow cytometry. Each test was repeated three times.

### Measurement of H_2_S concentration

H_2_S concentration in cell culture medium of cultured ECs was measured according to our previous report^55^. The cells were pretreated with ACh for 30 min at 37 °C. The cell culture medium was collected and incubated with 1% zinc acetate, 10% trichloroacetic acid, 20 µM *N*,*N*-dimethyl-p-phenylenediamine sulfate, and 30 µM FeCl_3_ for 25 min. The absorbance of the mixed solution was detected at 665 nm by a microplate reader. The H_2_S concentration was quantified according to the NaHS standard curve, which represents the amount of H_2_S released by ECs.

### Co-culture of cerebrovascular ECs with HNCs

Cell co-culture technique were in compliance with previous research with some modification^9^. Transwell co-culture system (Millicell small suspension cell culture insert, 0.4 µm polyester membrane, 6 wells; Corning, USA) was used to co-culture cerebrovascular ECs with HNCs for 24 h. The cells were pretreated with NaHS or ACh for 1 h before hypoxia to stimulate H_2_S release.

### Western blotting

The cells were lysated in radioimmunoprecipitation assay buffer, which consisted of Tris-HCl 50 mM, NaCl 150 mM, 1% sodium deoxycholate, 0.1% SDS, sodium fluoride, 1% Trition X-100, and EDTA. The total proteins were separated by 12% SDS-PAGE and then transferred to polyvinylidene difluoride membranes. The bands were visualized by using an enhanced chemiluminescence kit. Membrane protein and cytoplasmic protein extraction are performed according to the instructions in the kit. Briefly, the homogenate is centrifuged at a low speed to remove unbroken cells and nuclei, and the supernatant obtained by high-speed centrifugation contains cytoplasmic proteins. After the precipitate is dissolved, membrane proteins are obtained.

### Statistical analysis

All data in this study are expressed as mean ± SEM. The data analysis was carried out by a blinded investigator, who did not know which group the samples came from. Statistical analyses were performed to identify the normal distribution and homogeneity of variance of data by one-way analysis of variance, followed by Student’s *t*-tests to determine differences between groups. *P*-values < 0.05 were considered to be statistically significant.

## Supplementary information

Supplementary figure legends

Supplementary figure 1

Supplementary figure 2

Supplementary figure 3

Supplementary figure 4

Supplementary figure 5
