# Supplementary material for: H2S protects hippocampal neurons against hypoxia-reoxygenation injury by promoting RhoA phosphorylation at Ser188
Source: Cell Death Discov. 2021 Jun 4;7:132. doi: 10.1038/s41420-021-00514-z (PMC8178328; doi:10.1038/s41420-021-00514-z)
Supplement: Supplementary file 1 — Supplementary figure legends [file 41420_2021_514_MOESM1_ESM.docx]

**Supplementary figure legends**

**Figure S1. Plasmids digestion, sequencing, purification.** Digestion (A) and sequencing (B) of RhoA^wild^-pGEX-6p-1. Digestion (C) and sequencing (D) of RhoA^S188A^-pGEX-6p-1. (Lane M: DNA Marker; Lane 1: Plasmids digested BamHI XhoI Lane; 2: Plasmid DNA Digestion) (E) and sequencing (F) of RhoA^wild^ -pEGFP-N1. Digestion (G) and sequencing (H) of RhoA^S188A^-pEGFP-N1. (Lane M: DNA Marker Lane 1: Plasmids digested HindIII SacII Lane 2: Plasmids DNA) Sequencing results of coliform (BL-21) liquid (I). Coomassie brilliant blue staining and automatic exposure of PAGE plue for RhoA^wild^-pGEX-6p-1 (J) and RhoA^S188A^-pGEX-6p-1 (K). (1: Mark; 2: transfected with empty plasmids; 3: Uninduced; 4: Induced expression for 4h; 5. Supernatant; 6. Impurity protein 7. Purified protein)

**Figure S2. Identification of hippocampal neurons (HNCs) and endothelial cells.** (DAPI staining, 200μm) Immunofluorescence of HNCs incubated with microtubule-associated protein 2 (A–C) or phosphate balanced solution (PBS) (D–F). A positive fluorescence was detected in HNCs with the presence of microtubule-associated protein 2; not in the cells with the presence of PBS. Immunofluorescence of HNCs incubated with VIII factor (G–I) or PBS (J–L). The endothelial cells were captured positive fluorescence with the presence of VIII factor. DAPI was use to stain nucleus.

**Figure S3. Genetic identification of CSE^-/-^ and CSE^+/+^ mice as well as 3-MST^+/+^ and 3-MST^-/-^ rats.** (A) 369 bp represents CSE^-/-^; 167 bp refers to CSE^+/+^. (B) 521 bp represents 3-MST^-/-^;756 bp refers to 3-MST^+/+^.

**Figure S4. Effect of endothelial-derived H_2_S on RhoA phosphorylation, activity and translocation in neurons.** p-RhoA expressions (A), membrane fraction expression levels of RhoA (B), cytosolic fraction of RhoA (C) and RhoA activity (D) in HNCs, co-cultured with endothelial cells that were stimulated with ACh. E-cadherin and β-actin was used as a loading control for membrane and cytosolic proteins, respectively. Data are shown as the mean ± SEM; n= 3. **P*<0.05, ***P*<0.01.

**Figure S5. Effect of endothelial-derived H_2_S on ROCK_2_ protein expression and activity in neurons.** ROCK_2_ expression(A) and ROCK_2_ activity (B) in HNCs co-cultured with endothelial cells that were stimulated with ACh. β-actin was used as a loading control. (C) H_2_S concentration in endothelial cells derived from CSE^-/-^ mice and 3MST^-/-^ rats. Data are shown as the mean ± SEM; n = 3. **P*<0.05, ***P*<0.01.
