# Supplementary figures and images for: H2S protects hippocampal neurons against hypoxia-reoxygenation injury by promoting RhoA phosphorylation at Ser188
Source: Cell Death Discov. 2021 Jun 4;7:132. doi: 10.1038/s41420-021-00514-z (PMC8178328; doi:10.1038/s41420-021-00514-z)

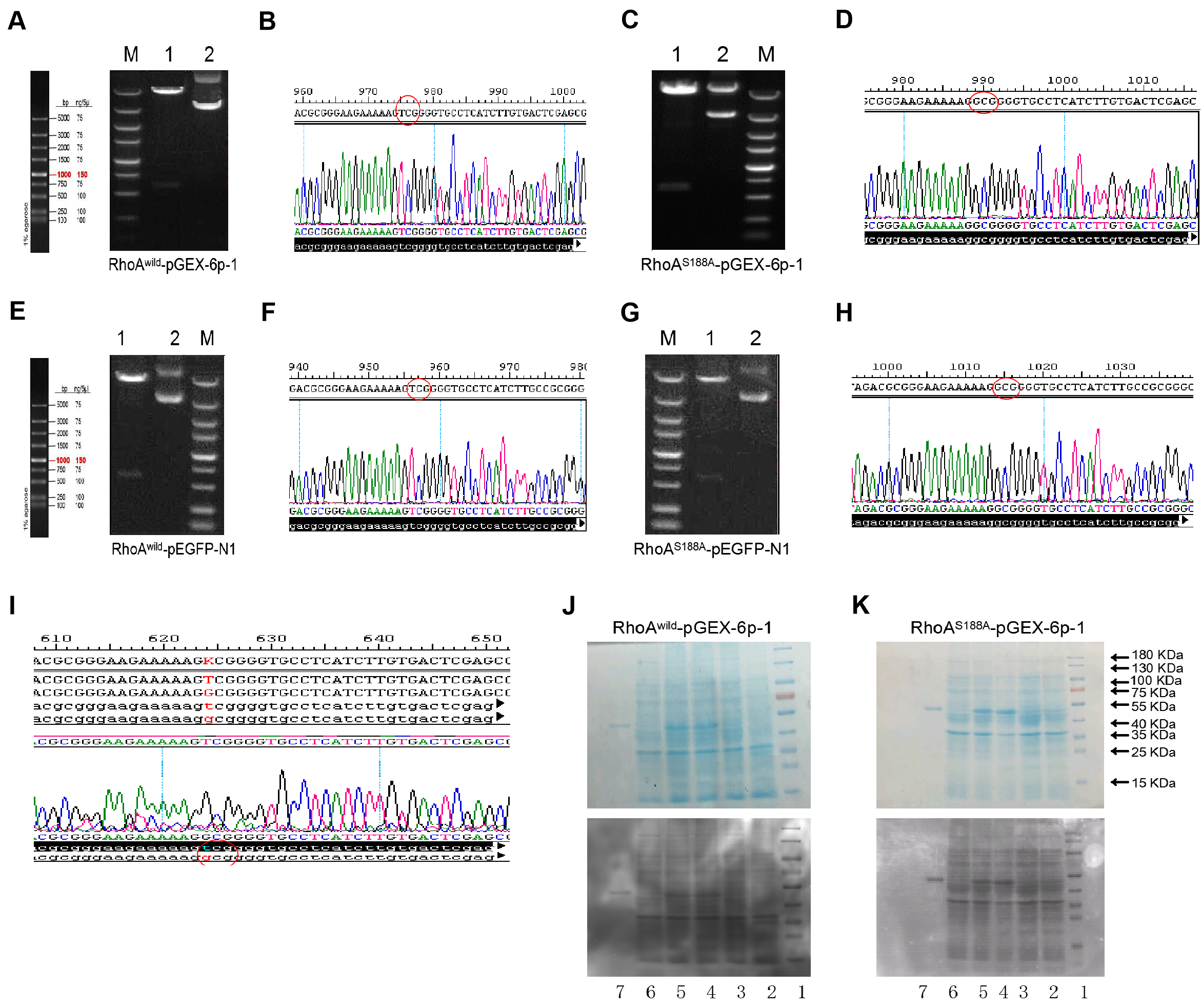

Supplement: Supplementary file 2 — Supplementary figure 1 [file 41420_2021_514_MOESM2_ESM.tif]

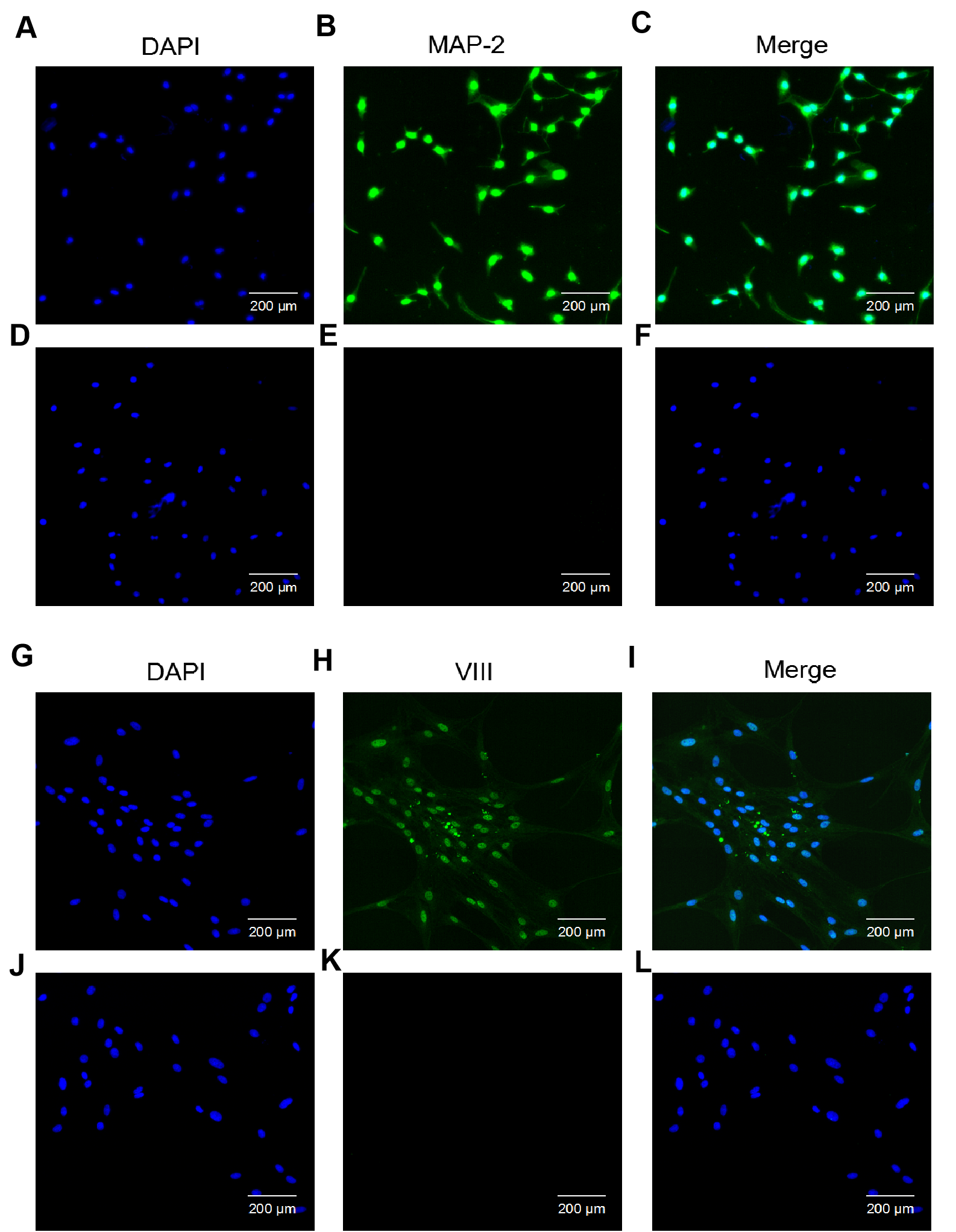

Supplement: Supplementary file 3 — Supplementary figure 2 [file 41420_2021_514_MOESM3_ESM.tif]

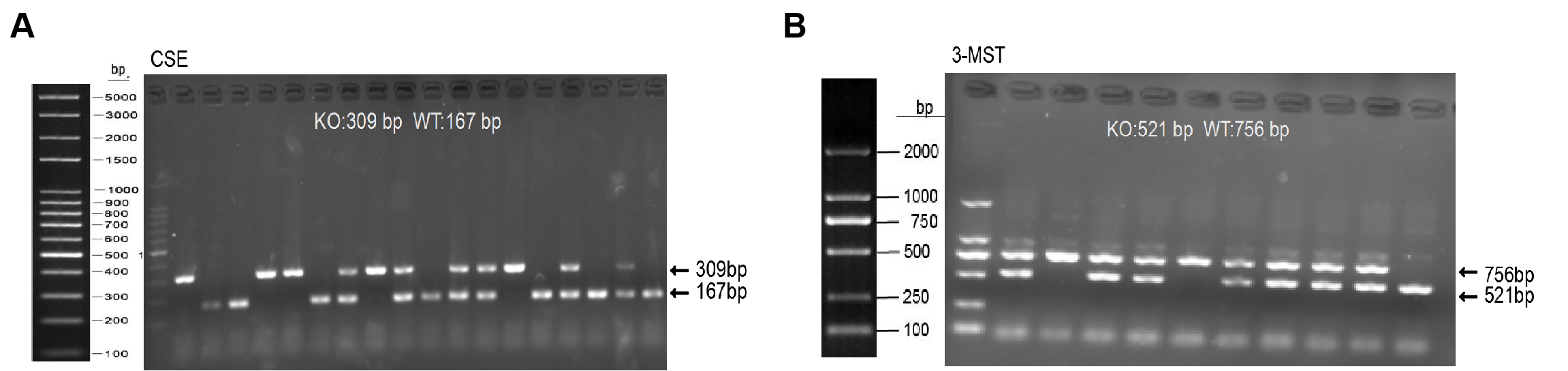

Supplement: Supplementary file 4 — Supplementary figure 3 [file 41420_2021_514_MOESM4_ESM.tif]

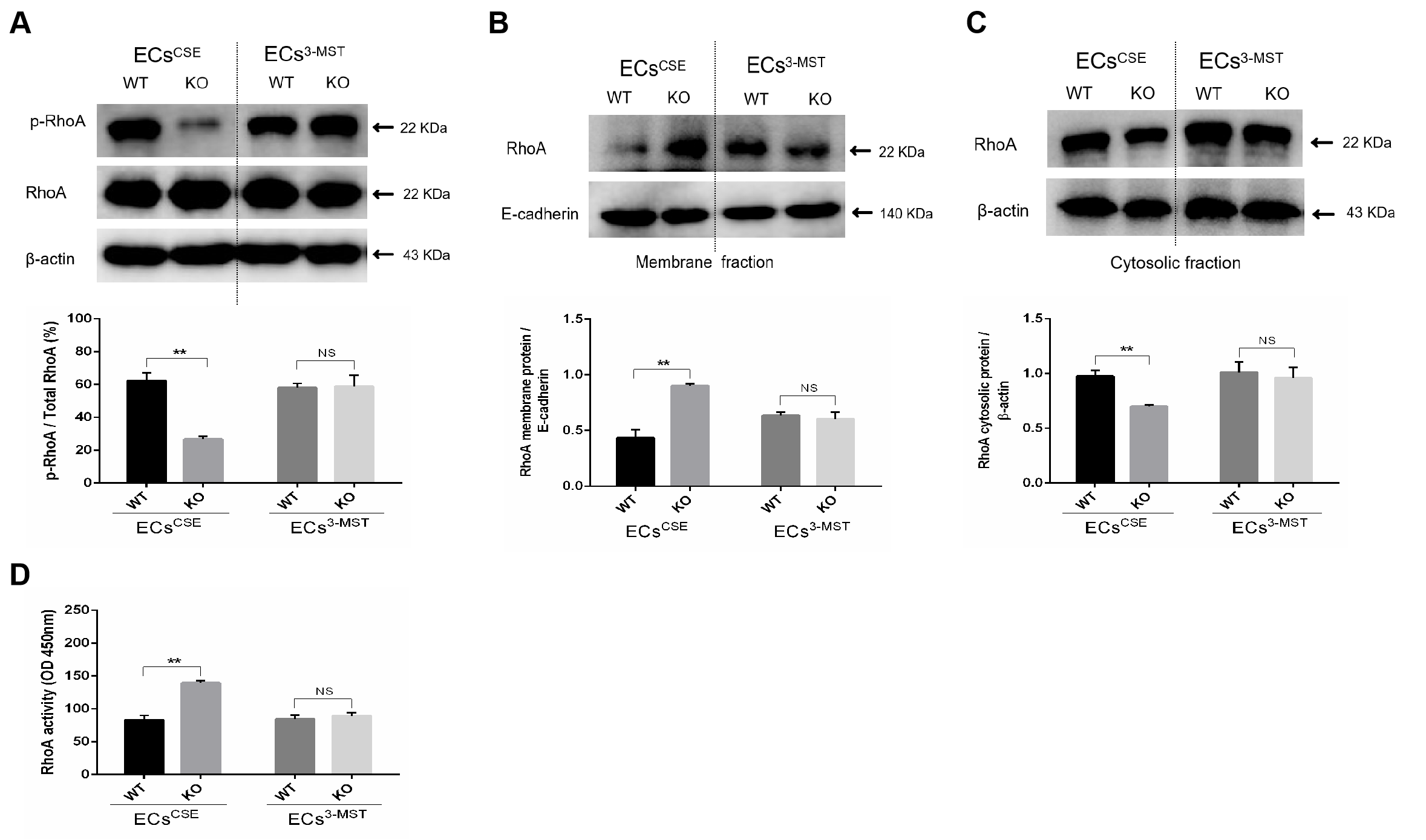

Supplement: Supplementary file 5 — Supplementary figure 4 [file 41420_2021_514_MOESM5_ESM.tif]

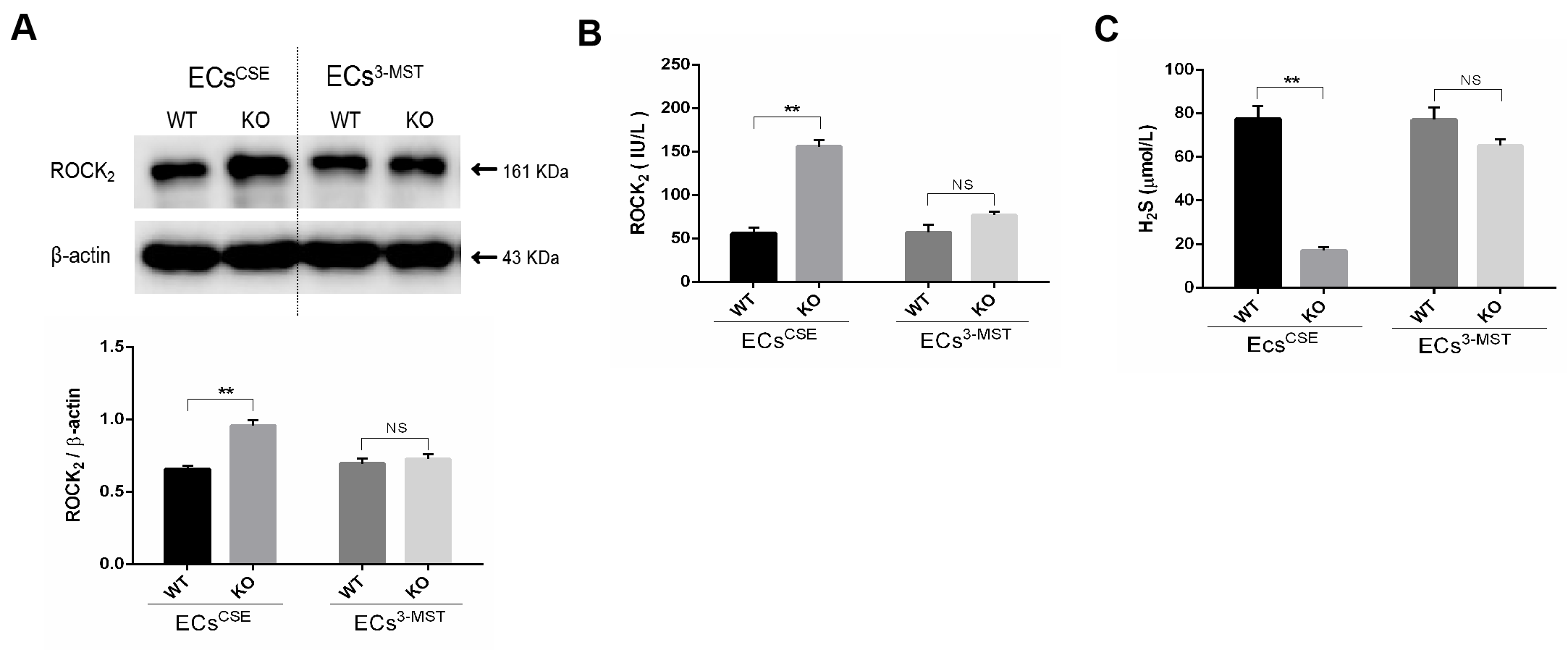

Supplement: Supplementary file 6 — Supplementary figure 5 [file 41420_2021_514_MOESM6_ESM.tif]
